# Characterising the protective vasodilatory effects of hypobaric hypoxia on the neurovascular coupling response

**DOI:** 10.1177/0271678X251322348

**Published:** 2025-03-13

**Authors:** Jack K Leacy, David P Burns, Nicholas G Jendzjowsky, Connor Braun, Brittney A Herrington, Richard JA Wilson, Tyler D Vermeulen, Glen E Foster, Alexander J Rosenberg, Garen K Anderson, Caroline A Rickards, Eric F Lucking, Ken D O’Halloran, Trevor A Day

**Affiliations:** 1 Department of Physiology, 8795 School of Medicine, College of Medicine and Health, University College Cork, Cork, Ireland; 2 Hotchkiss Brain institute, University of Calgary, Calgary, Alberta, Canada; 3 Respiratory and Critical Care Medicine and Physiology, The Lundquist Institute for Biomedical Innovation at Harbor-UCLA Medical Centre, Torrance, CA, USA; 4 Physiology and Pharmacology, Cumming School of Medicine, University of Calgary, Calgary, Alberta, Canada; 5 School of Health and Exercise Sciences, Faculty of Health and Social Development, University of British Columbia Okanagan, British Columbia, USA; 6 Cerebral and Cardiovascular Physiology Laboratory, School of Biomedical Sciences, University of North Texas Health Science Centre, Texas, USA; 7 Physiology Department, Midwestern University, Dower Grove, IL, USA; 8 Department of Biology, Faculty of Science and Technology, Mount Royal University, Calgary, Alberta, Canada

**Keywords:** Cerebral blood flow, high altitude, acclimatization, hypobaric hypoxia, neurovascular coupling, transcranial Doppler ultrasound

## Abstract

Neurovascular coupling (NVC) is the link between local neuronal activity and regional cerebral blood flow. High altitude (HA) ascent induces acute hypoxic vasodilation of the cerebral vasculature, with associated changes in CO_2_ and acid-base status. We aimed to characterise the effects of (a) acute removal of the HA-induced vasodilation and (b) rapid ascent to and residency at HA on NVC responses. In twelve healthy participants (7 M/5F), arterial blood gases and NVC were measured at baseline (1130 m) and on days two (<24 h at HA) and nine (post-acclimatisation) at 3800 m. Acute gas challenges were performed using end-tidal forcing, with (a) normoxia and isocapnic hypoxia at 1130 m and (b) poikilocapnic hypoxia and isocapnic hyperoxia on days two and nine at 3800 m. Posterior cerebral artery velocity (PCAv) was measured using transcranial Doppler ultrasound in each condition and time-point. NVC was assessed via a standardized 30 s intermittent strobe light visual stimulus (VS), and quantified as the peak and mean change from baseline in PCAv. No significant differences were observed for any NVC metric across all conditions and time points. Our results reveal remarkable stability of the NVC response following (a) acute removal of HA-induced hypoxic vasodilation and (b) rapid ascent to and residency at 3800 m.

## Introduction

High altitude exposure has a time-dependent effect on cerebrovascular function. Following acute exposure to high altitude, there is an immediate hypoxemia-mediated cerebral vasodilation and increase in global cerebral blood flow.^1–11^ This relative increase in cerebral blood flow serves to maintain cerebral oxygen delivery during instances of arterial hypoxemia.^3,5,11–14^ With time spent at high altitude, there is a progressive reduction in global cerebral blood flow to values comparable with residency altitude.^1,2,5,6,8–11,15,16^ The time-dependent reduction in cerebral blood flow occurs concomitantly with polycythemia, cerebrospinal fluid pH buffering, alterations in hypoxia-inducible factors (HIF) expression, cerebral angiogenesis, hyperventilation-induced increases in PaO_2_ and consequent reductions in PaCO_2_.^1,5,9,10,16^ While these observations support altitude-induced changes in global cerebral blood flow, they provide little insight into the effect of HA exposure on regional control of cerebral blood flow.

Neurovascular coupling (NVC) represents the link between local neuronal activity and regional cerebral blood flow.^17–20^ NVC ensures focal changes in cerebral metabolism are met with increases in the delivery of glucose and O_2_ to the active region, supporting energy-dependent metabolic processes. Cerebrovascular tone is sensitive to a milieu of metabolic and chemical stimuli. Notably, alterations in arterial PO_2_ and PCO_2_ as well as intra- and extracellular pH conditions can influence cerebrovascular smooth muscle cells and endothelium, with subsequent effects on basal vessel calibre, and vasomotion. Acidotic and alkalotic conditions induce vasodilation and vasoconstriction of cerebral vessels, respectively.^21–28^ Blood gas changes and transient disruption of acid-base status are consistently observed with high altitude exposure. The degree to which high altitude -induced disruption in arterial blood gases and acid-base homeostasis affects NVC is still poorly characterised.

To our knowledge only three previous studies have examined the effects of high altitude exposure on NVC in human participants. Two of these publications were conducted by members of our research group in the Nepal Himalaya,^29,30^ while the other was performed at the Barcroft laboratory in California.^31^ Collectively, these studies demonstrated that NVC was unaffected by exposure to high altitude. However, owing to the delay between initial high altitude exposure and NVC assessment, in combination with the ascent profile(s) employed, the likelihood is that all participants were fully acclimatised at the point of measurement. Additionally, only one of these studies performed arterial blood gas analysis, confirming normalised arterial pH during incremental ascent to 4240 m,^29^ indicative of acclimatisation to high altitude. As such, these studies confirm that NVC remains stable at high altitude in the background of normalised pH but provide little insight into the extent of respiratory-induced pH changes at high altitude on NVC. More importantly, these studies did not characterise the degree to which hypoxic vasodilation might preserve NVC function at HA, against the background of multiple vasoconstrictive stressors (i.e., hypocapnia, respiratory-induced alkalosis).

We aimed to answer two key questions: 1) with acute (<24 h) high altitude exposure, does the competing hypoxic vasodilatory stimulus offset any hypocapnic/alkalotic-dependent NVC impairment? and 2) does respiratory-induced alkalosis following acute exposure to high altitude induce NVC impairment? We hypothesized that: 1) removal of the vasodilatory hypoxic stimulus following acute exposure to HA would impair NVC magnitude and 2) acute exposure to HA would impair NVC, which would return to normal following acid-base acclimatisation.

## Material and methods

### Ethical approval & participant recruitment

This study abided by research guidelines and policy concerning human participants set out by the Canadian Government Tri-Council and conformed to the latest standards set by the Declaration of Helsinki, except for registration in a database. Ethical approval was received in advance through the University of Calgary Conjoint Human Research Ethics Board (Protocol #REB18-0374) and harmonized with the Mount Royal University Human Research Ethics Board (Protocol #101879).

Participants were recruited from a large research expedition which started in Calgary, Alberta, Canada (1130 m) for baseline testing, then travelled to the Barcroft Lab located at 3800 m near White Mountain Summit in California, USA. Although there is some overlap with participants included in this study and other previously published studies from the same expedition (Bird et al., 2021; Baker et al., 2022), the blood gas and acid-base acclimatization data utilized here are from a subset of participants (n = 12) linked to NVC assessment with ascent to and residence at 3800 m. Thus, the study conducted and reported here represents an *a priori* investigation on a novel research question.

**Figure 1.**
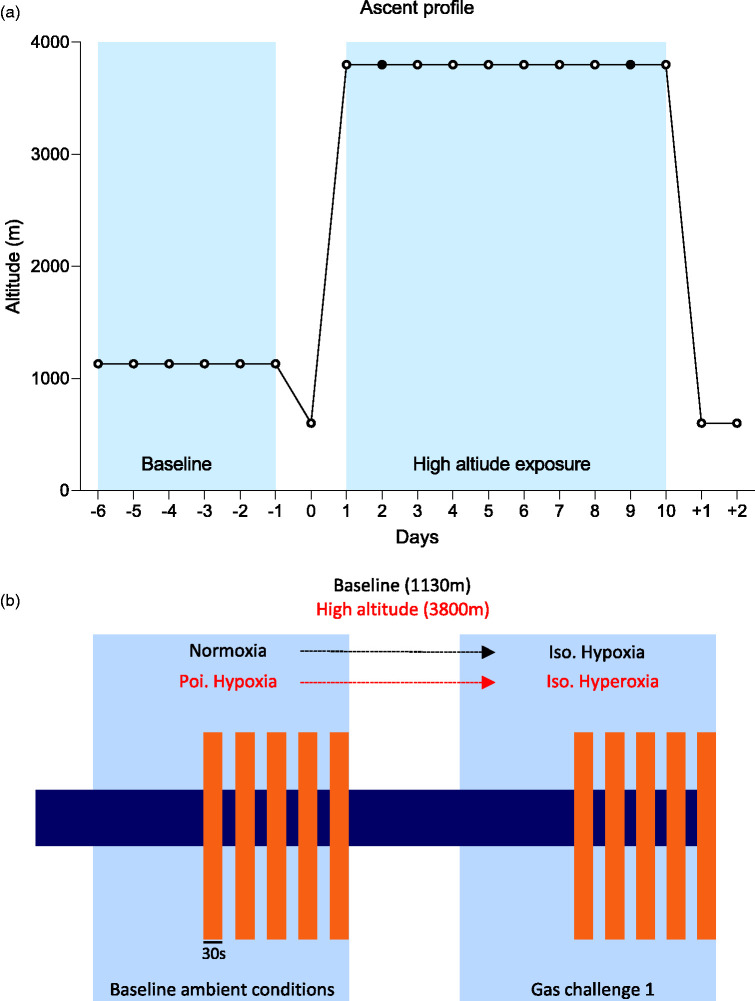
Ascent profile and study methodology. Illustrations of the ascent profile & inherent study methodology used within this research expedition are provided (A and B, respectively). (a) Baseline measures were collected over a five day period at 1130 m (Calgary, Canada) followed by an overnight stay at 610 m (Las Vegas, Nevada). Participants sojourned by car from 610 m to 3800 m (Barcroft research station, California, USA) over a 5–6 hour period. Measurements were taken on day 2 and 9 of high altitude exposure (3800 m; black dots) before returning to Las Vegas and (b) An illustration of the precise methodology employed is provided. Prevailing gas conditions for sea-level and high altitude test days are provided in black and red, respectively. Orange shaded boxes denote periods of 30 second visual stimulation.

Twelve healthy participants (7 males, 5 females, 28.7 ± 8.7yrs, 72.1 ± 13.5 kg, 24.1 ± 3.0 kg/m^2^) were recruited and provided informed verbal and written consent prior to voluntary participation. Previously published work from our research group has shown that neither age nor sex affect the NVC response of the PCA.^32^ Therefore, demographic heterogeneity in age and sex profile within this sample population likely does not cofound group NVC assessment across stimuli. Prior to data collection, a medical pre-screening questionnaire was completed and reviewed to exclude participants with pre-existing contraindications. Participants were excluded if they reported any prior or current medical history of neurological, cardiovascular, cerebrovascular and/or pulmonary disease. All baseline data were collected at Mount Royal University in Calgary (1130 m), over a five-day period prior to high altitude ascent. After which, participants flew to Las Vegas (610 m) where they spent one night. This expedition employed a rapid ascent profile relative to previously published work from our research group.^29,33^ Ascent time to the Barcroft research station (3800 m) was approximately 5–6 h from Las Vegas by car, facilitating acute, rapid exposure to hypobaric hypoxia. High altitude data collection was conducted on day two (≤24 h high altitude exposure but following one night sleep at 3800 m; WM2) and day nine of high altitude residency (WM9; see Figure 1(a)).

### Instrumentation

Participants were instrumented and tested in a seated position. Beat-by-beat measurement of heart rate (bpm) and arterial blood pressure (systolic/diastolic/mean arterial pressure; mmHg) were measured using an electrocardiogram (ECG: lead II configuration; Bioamp ML132; AD Instruments, Colorado Springs, CO, USA) and finometer (Finapres Medical Systems, Amsterdam, NL), respectively. Participants were instrumented with a mouthpiece, nose clip and a two-way valve system, the inspiratory port of which was open to either ambient air or an end-tidal forcing system (Airforce, Pneumologix Consulting Ltd., Kelowna, BC, Canada). The forcing system controls inspiratory O_2_ and CO_2_ gas mixtures by a feedback control and error correction algorithm to achieve targeted end-tidal values. The end-tidal forcing system was calibrated on the morning of each test day. A calibrated pneumotachometer, attached proximal to the two-way valve, was used to measure respiratory flow (HR 800 L flow head and spirometer amplifier, ML141, AD Instruments, Colorado Springs, CO, USA). Inspiratory minute ventilation (V̇_I_; L/min) was calculated as the product of breath-by-breath inspiratory tidal volume (V_TI_: Litres) and respiratory rate (R_R_; min^−1^). Peripheral oxygen saturation was measured using a portable finger pulse oximeter secured to the right index finger (SpO_2,_ %; Oximeter pod and sensor, ML320, AD Instruments, Colorado Springs, CO, USA).

Beat-by-beat cerebral blood velocity was measured in the posterior cerebral and middle cerebral artery (PCAv and MCAv respectively; cm/s) using a transcranial Doppler ultrasound (TCD; Spencer technologies, Redmond, WA, USA). Beat-by-beat measurements of cerebrovascular conductance (CVC) and pulsatility index (PI) were continuously recorded for the MCA and PCA vessels. Where possible, the P2 segment of the PCA was insonated as the perfusion territory of this vessel segment is more proximal to the downstream neuronal pool involved in visual processing, compared with the P1 segment.^34^ Insonation of intracranial arteries was performed using previously described methods employed by our group.^29,32,35^ A headpiece was used to affix both 2 MHz Doppler ultrasound probes on either side of the cranium, insonating through the trans-temporal acoustic window. The side of the cranium and depth of each insonated vessel was recorded at baseline (1130 m) and replicated on days two and nine at 3800 m. Beat by beat assessment of regional cerebral oxygen saturation (S_c_O_2_; %) within the frontal cortex was measured using near infrared-spectroscopy (NIRS; INVOS cerebral/somatic oximeter; Somanetics Corporation, Michigan, USA). S_c_O_2_ was measured on the ipsilateral hemisphere of the insonated MCA. An S_c_O_2_ measurement was hand recorded every thirty seconds during each ventilatory challenge and averaged to provide a mean S_c_O_2_ for each challenge.

Arterial blood draws were obtained on one day during the baseline testing period (not necessarily the day of NVC assessment), and on days two and nine at high altitude (WM2 and WM9). Samples were obtained from the radial artery and analysed immediately using a portable blood gas analyser (Abbott i-STAT, CG4+ and CHEM8+ cartridges; Mississauga, Ontario). Arterial blood sampling allowed for precise measurement of arterial oxygen saturation (SaO_2_), partial pressure of arterial oxygen and carbon dioxide (P_a_O_2_ and P_a_CO_2_, respectively; mmHg), arterial bicarbonate concentration ([ ${\text{HCO}}_{3}^{-}$ ], mmol/L), base excess (mmol/L), arterial pH, hematocrit (%) and hemoglobin concentration (g/L). For precise measurement, blood gases were corrected for body temperature and local atmospheric pressure. Arterial blood gases and pH measures were used to infer oxygenation and acid-base acclimatisation status within each participant.^36^

## Experimental protocol

NVC was assessed in the background of two distinct ventilatory conditions: (a) inspiration of ambient air, and (b) inspiratory gas challenge (specific to location, see Figure 1(b)). Inspiration of ambient air constitutes a normoxic stimulus at baseline (Patm = 670 mmHg, Inspired PO_2_ = 139 mmHg) and a poikilocapnic hypoxic (Patm = 487 mmHg, Inspired PO_2_ = 99) stimulus at high altitude, respectively. At baseline, the inspiratory gas challenge was an isocapnic-hypoxic stimulus (P_atm_ = 490 mmHg, inspired PO_2_ = 103 mmHg). The purpose of this stimulus was to match P_ET_O_2_ to levels anticipated at Barcroft research station (3800 m) by manipulation of inspired PO_2_. In contrast, the inspiratory gas challenge on days two and nine at 3800 m was an isocapnic-hyperoxic challenge. Again, the relative hyperoxic gas mixture served to mimic P_ET_O_2_ levels observed at baseline (1130 m) by manipulation of inspired PO_2_. Exposure to the hyperoxic gas was designed to acutely alleviate the hypoxic stimulus of high altitude and return participants to an oxygenation value similar to that observed during normoxia at baseline (1130 m). The precise procedural and temporal aspects of the experimental protocol used during each ventilatory challenge are detailed below and shown in Figure 1(b).

Participants completed an initial 3-min baseline period, in the seated position, inspiring ambient air (without end-tidal forcing). Participants were instructed to keep their eyes closed for the duration of this period. Upon completion of baseline, participants were exposed to approximately 180 seconds of optical coherence tomography 3D retinal imaging (data not shown; separate published investigation^37^) Following completion of optical coherence tomography imaging, and the establishment of a resting baseline, participants were instructed to close their eyes for a 1-min epoch immediately prior to NVC assessment. NVC was assessed using five repeated trials of intermittent visual stimulation (30 s on/off visual stimulation). The visual stimulus was elicited using an intermittent strobe light stimulus on a mobile phone app (6 Hz; *Strobe* app). Participants were verbally queued when to open/close their eyes with a researcher confirming adherence to verbal instructions. This method of inducing an NVC response has been previously used by our group without any adverse effects to the strobe light stimulus.^29,32,35^ Upon completion of visual stimulus trials, participants were instructed to sit for a further 30 s with their eyes closed. The completion of this 30 s epoch signalled the end of the NVC test protocol for each respective gas challenge. This exact protocol was repeated for each ventilatory challenge on each test day. During the isocapnic hypoxic and hyperoxic gas challenges, the three-min baseline period did not begin until steady-state control of end-tidal gases was achieved (≈60 s).

## Data analysis protocol

**Figure 2.**
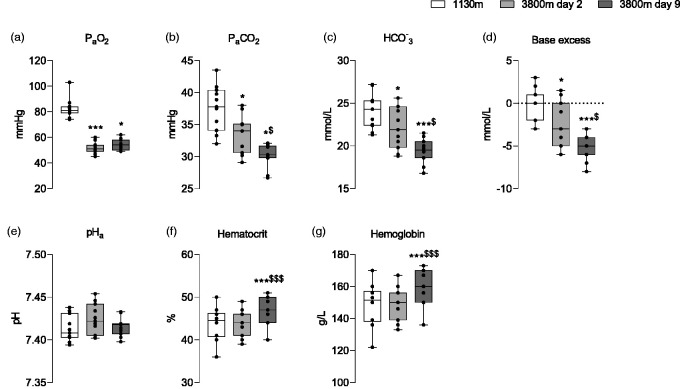
Arterial blood draw data across locations. Measurements of arterial oxygen concentration (PaO_2_; a), arterial carbon dioxide concentration (PaCO_2_; b), arterial bicarbonate ( ${\text{HCO}}_{3}^{-}$ ; c), base excess (d), arterial pH (e), Hematocrit (f) and Hemoglobin (g) are provided across test days. Measurements for 1130 m, 3800 m day 2 and 3800 m day 9 are provided in clear, light grey and dark grey boxes respectively. Data is presented in boxplot format showcasing mean ± SD. */*** = p < 0.05/<0.001 from 1130 m respectively; ^$/$$$^= p < 0.05/<0.001 from 3800 m day 2 respectively.

**Figure 3.**
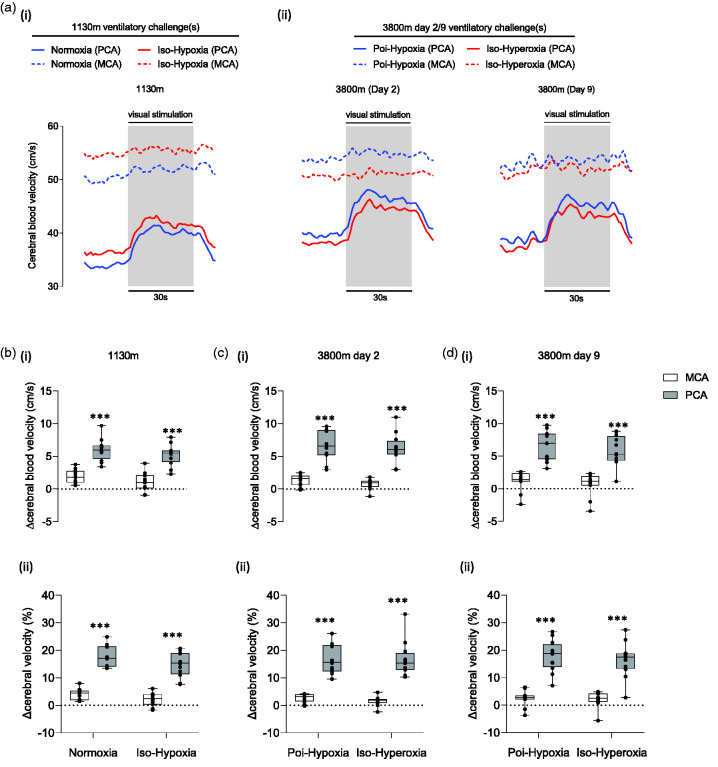
Spatial specificity of the visually-evoked hemodynamic response. (a) Group averaged waveforms during visual stimulation (grey region) for middle and posterior cerebral arteries (MCA/PCA respectively) are provided for each gas challenge at 1130 m (i) and 3800 m (ii). Cerebral velocity is presented along the y-axis. MCA waveforms are presented as dotted lines. PCA waveforms are presented as continuous lines. (b–d) The magnitude of the hemodynamic response, within the MCA (clear boxes) and PCA (grey boxes), to visual stimulation is presented. Experimental conditions are presented along the x-axes. Y-axes represent the magnitude of the response as a change in absolute units (Δcm/s; top row) and as a relative change from baseline (Δ%; bottom row). Data is presented in boxplot format with mean ± SD. *** = P < 0.001 from gas-matched MCA.

**Figure 4.**
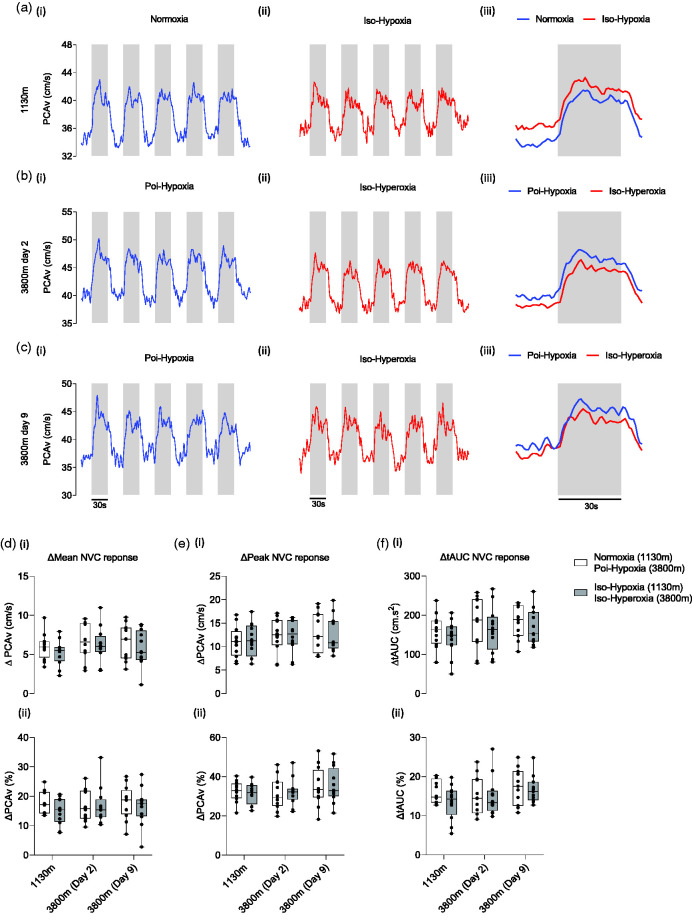
Neurovascular coupling waveform(s) and response magnitude between experimental conditions. (a–c) Group averaged waveforms of the posterior cerebral artery velocity (PCAv) during each visual stimulus (a-ci-ii), and the average PCAv response during visual stimulus (a-ciii), are presented for each experimental condition. PCAv is presented along the y-axes. (d–f) The magnitude of the hemodynamic response to visual stimulation is presented for each NVC metric; ΔMean (di-ii); ΔPeak (ei-ii); ΔtAUC (fi-ii). Test locations are presented along the x-axes. Y-axes represent the magnitude of the response as a change in absolute units (Δcm/s; top row) and as a relative change from baseline (Δ%; bottom row). Data is presented in boxplot format with mean ± SD.

**Figure 5.**
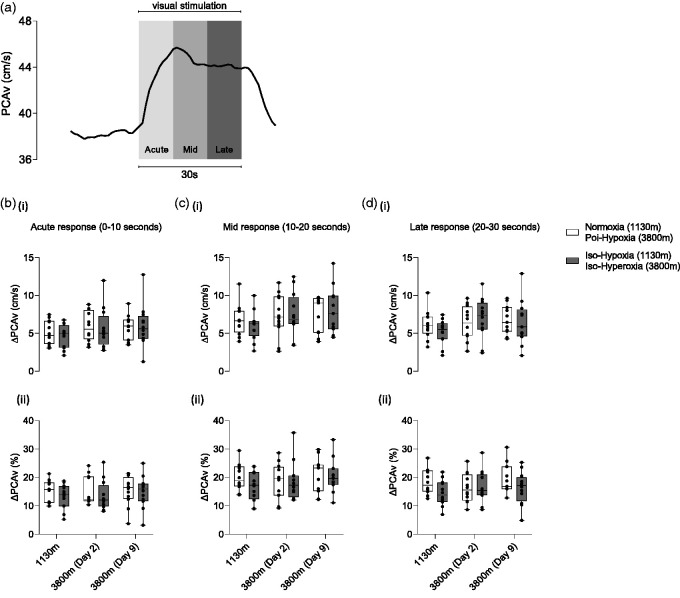
Temporal-dependent regional analysis of the neurovascular coupling response across experimental conditions. (a) Group averaged hemodynamic response within the posterior cerebral artery during visual stimulation. Distinct temporal regions of the hemodynamic response, relative to the onset of visual stimulation, are illustrated. PCAv is presented along the y-axis. (b–d) The magnitude of the hemodynamic response to visual stimulation is presented for each temporal region; ΔAcute (bi-ii); ΔMid (ci-ii); ΔLate (di-ii). Test locations are presented along the x-axes. Y-axes represent the magnitude of the response as a change in absolute units (Δcm/s; top row) and as a relative change from baseline (Δ%; bottom row). Data is presented in boxplot format with mean ± SD.

**Figure 6.**
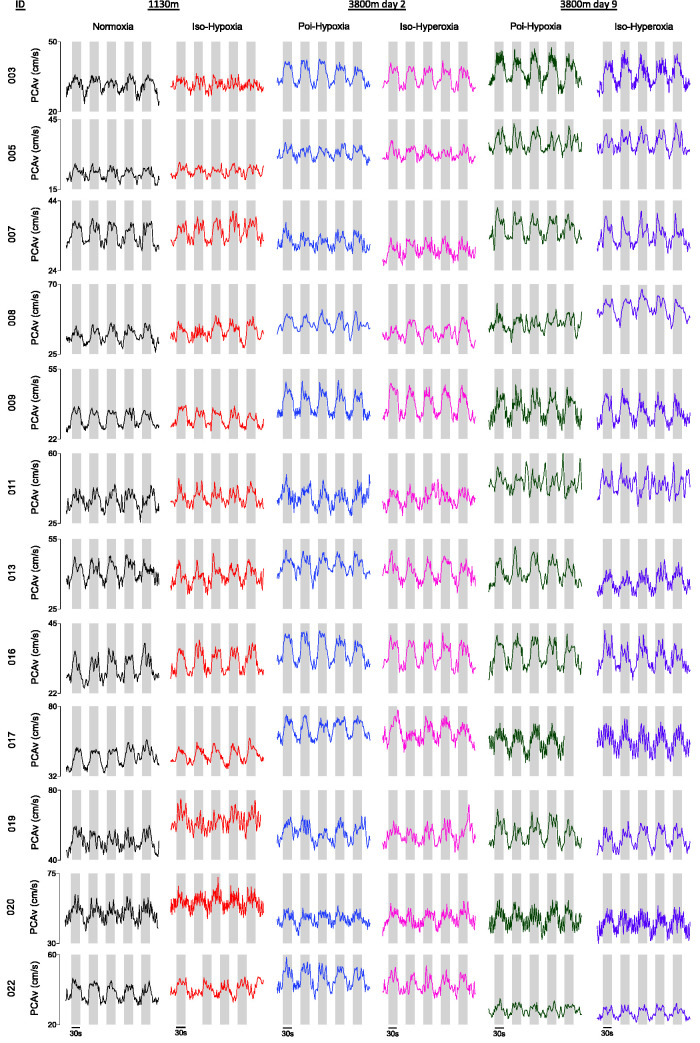
Individual posterior cerebral artery velocity (PCAv) waveforms during repetitive visual stimulation across all conditions. An illustration of the individual NVC response within the PCA is provided for all experimental conditions. Grey shaded regions denote 30-second periods of visual stimulation. Participant ID and PCAv are presented along the y-axes. Intra-individual y-axis ranges are consistent across experimental conditions. Experimental conditions are grouped according to test location (1130 m, 3800 m day 2 and day 9).

Data acquisition was completed using Labchart software (v8.0) with resultant figures developed using Graphpad prism 8 software. Individual and group averaged PCAv and MCAv waveforms (Figures 3 to 6) across all experimental conditions were derived using a custom macro on Labchart. All baseline cardiorespiratory and cerebrovascular measures (Table 1) were obtained by calculating 1-min averages immediately prior to NVC assessment across each experimental condition. Arterial blood data were derived from point measurements taken throughout each testing day (Figure 2).

**Table 1.** Participant baseline cardiorespiratory and cerebrovascular measures across experimental conditions. Group-averaged baseline values for cardiorespiratory and cerebrovascular variables are provided for each experimental condition. Experimental conditions are grouped according to test location (Calgary [1130 m]; White Mountain [3800m] day 2 or 9). Data which were normally distributed are presented as mean ± SD. In contrast, data which violated normal distribution are presented as median (25^th^-75^th^ percentile). P-values for repeated measures analysis of variance (parametric data) or Friedman ranks test (non-parametric data) are provided. Effect sizes refer to partial eta squared (N_p_^2^; parametric data) or Kendall’s W (*W*; non-parametric data). * = p < 0.05 from Normoxia; ^&^ = p < 0.05 from Iso-Hx (1130 m); ^%^ = p < 0.05 from Poi-Hx (White Mountain day 2); ^$^= p < 0.05 from Iso-Hyperoxia (White Mountain day 2); ^@^ = p < 0.05 from Poi-Hx (White Mountain day 9).

Baseline cardiorespiratory and cerebrovascular measures								
	Calgary		White Mountain Day 2		White Mountain Day 9		One-way RM ANOVA/Friedman test	
	Normoxia (n = 12)	Iso-Hx (n = 12)	Poi-Hx (n = 12)	Iso-Hyperoxia (n = 12)	Poi-Hx (n = 12)	Iso-Hyperoxia (n = 12)	p-value	Effect size
Cardiorespiratory
HR (bpm)	77.06 (66.18–84.92)	83.73 (78.30–90.56)*	81.89 (77.66–91.62)*	78.25 (75.03–87.79)	82.95 (75.24–92.81)	77.74 (76.45–92.27)	0.023	0.218
MAP (mmHg)	94.98 (89.35–109.19)	100.84 (97.22–114.71)	108.21 (98.90–118.01)*	107.29 (95.64–116.84)*	106.89 (95.64–116.84)	103.49 (90.49–117.59)	0.003	0.297
SBP (mmHg)	129.19 (121.21–145.72)	136.96 (132.51–154.52)*	138.89 (132.63–156.41)	141.81 (128.59–157.36)	135.36 (126.72–149.88)	131.36 (122.73–154.10)	0.003	0.298
DBP (mmHg)	77.07 ± 10.10	81.86 ± 10.54*	88.98 ± 11.44*	89.33 ± 11.26*	86.79 ± 9.96	84.56 ± 11.72	0.005	0.376
R_R_ (min^−1^)	15.55 (12.86–17.84)	17.80 (15.01–19.76)	16.47 (12.37–19.72)	14.49 (13.26–15.62)	14.42 (11.82–19.59)	15.29 (12.65–18.09)	0.330	0.096
V̇_TI_ (L)	0.95 ± 0.35	1.22 ± 0.53*	1.72 ± 0.54*	1.88 ± 0.70*	1.85 ± 0.54*^&^	1.89 ± 0.72*	0.001	0.427
V̇_I_ (L/min)	15.55 (7.86–18.10)	20.74 (11.93–28.88)	29.39 (19.94–33.00)*	30.02 (19.58–34.31)*	26.77 (19.57–32.01)*	28.69 (20.26–29.85)*	0.002	0.324
SpO_2_ (%)	97.70 (96.89–98.20)	86.13 (84.63–87.14)*	88.36 (84.59–89.37)*	97.93 (97.42–98.44)^&%^	89.24 (86.88–91.06)^$^	97.44 (95.50–98.34)^&%^	<0.001	0.853
P_ET_O_2_ (mmHg)	87.79 (84.18–90.70)	51.59 (51.05–51.91)*	52.49 (51.63–54.88)*	85.94 (80.33–93.09)^&%^	55.42 (53.86–58.19)*	85.22 (82.05–90.34)^&%^	<0.001	0.853
P_ET_CO_2_ (mmHg)	36.10 (33.82–38.53)	35.74 (33.56–39.68)	31.60 (30.06–33.45)^&^	32.53 (31.30–35.09)	29.21 (27.67–30.30)*^&$^	30.36 (28.39–31.08)*^&^	<0.001	0.818
Cerebrovascular
MCAv (cm/s)	50.63 ± 13.12	57.52 ± 19.71	55.66 ± 11.15	52.53 ± 9.19	54.51 ± 11.24	55.46 ± 11.62	0.488	0.068
MCA_CVC_	0.52 ± 0.13	0.54 ± 0.15	0.51 ± 0.11	0.48 ± 0.08	0.53 ± 0.10	0.55 ± 0.11	0.472	0.072
MCA_PI_	1.00 ± 0.12	0.92 ± 0.12	0.83 ± 0.13*	0.83 ± 0.13*	0.93 ± 0.18	0.88 ± 0.16	0.014	0.334
MCAv_Sys_	84.44 ± 24.08	90.96 ± 29.20	85.70 ± 17.29	80.58 ± 14.95	89.26 ± 14.75	87.29 ± 15.94	0.446	0.075
MCAv_Dia_	33.61 ± 7.86	38.71 ± 12.54	39.77 ± 9.12	37.32 ± 7.17	38.97 ± 8.38	39.63 ± 8.74	0.315	0.109
PCAv (cm/s)	37.25 ± 8.55	40.88 ± 11.72	44.33 ± 8.11	37.67 ± 13.56	42.39 ± 8.08	42.86 ± 8.04	0.257	0.116
PCA_CVC_	0.38 ± 0.08	0.38 ± 0.09	0.40 ± 0.05	0.38 ± 0.06	0.40 ± 0.05	0.42 ± 0.06	0.392	0.080
PCA_PI_	0.94 (0.84–1.05)	0.83 (0.79–1.08)	0.75 (0.71–0.88)*	0.75 (0.72–0.86)*	0.79 (0.69–1.09)	0.79 (0.65–0.96)	0.005	0.276
PCAv_Sys_	60.53 ± 12.42	63.65 ± 14.92	66.00 ± 10.41	62.28 ± 11.78	65.98 ± 12.93	65.47 ± 12.67	0.404	0.081
PCAv_Dia_	24.96 ± 5.86	27.46 ± 7.96	31.77 ± 5.54*	30.08 ± 5.78	29.93 ± 5.15	30.73 ± 5.59	0.018	0.284
S_c_O_2_ (%)	72 (70–77.5)	65.4 (63.33–70)	66.85 (58.79–69.75)	76.37 (68.75–80)^&%^	64.63 (58.63–70.63)^$^	76.5 (68.5–78)^&%@^	<0.001	0.704

* = p < 0.05 from Normoxia; ^&^ = p < 0.05 from Iso-Hx (Calgary); ^%^ = p < 0.05 from Poi-Hx (White Mountain day 2); ^$^ = p < 0.05 from Iso-Hyperoxia (White Mountain day 2); ^@^ = p < 0.05 from Poi-Hx (White Mountain day 9).

To demonstrate the anatomical specificity of the cerebral hemodynamic response during VS, the MCAv NVC response during visual stimulus was used as a “negative control”. The perfusion territory of the MCA is not associated with visual integration and processing and as such should not be responsive to visual stimulus with only minor fluctuations in cerebral velocity observed owing to resting cardiorespiratory and autonomic variability. The difference (Δ) in mean MCAv (cm/s; Δmean) achieved during visual stimulus exposure, relative to a 20 s epoch immediately prior to visual stimulus onset was calculated and compared with the PCA. The magnitude of the MCAv NVC response (Δmean) was measured and presented as an absolute change (Δcm/s) and relative change from baseline (Δ%) (Figure 3).

NVC was measured in accordance with previously published work from our group.^29,32^ NVC was quantified as the difference (Δ) in mean and maximum PCAv (Δcm/s; Δmean and Δpeak, respectively) achieved during visual stimulus exposure, relative to a 20 s epoch immediately prior to VS onset. We further quantified NVC as the difference (Δ) in total area under the curve (Δcm.s^2^; ΔtAUC) of the raw PCAv signal, relative to a 30 s epoch immediately prior to visual stimulus onset. The 30 s baseline period for tAUC analysis was used to ensure time-equivalency between baseline and visual stimulus periods when making total area comparisons. The magnitude of the NVC response for each parameter (Δmean, Δpeak and ΔtAUC) is presented as an absolute change (Δcm/s and Δcm.s^2^, respectively) and relative change from baseline (Δ%) (Figure 4). Moreover, the NVC response was sub-compartmentalised into three distinct post-stimulus epochs: acute (0–10 seconds), mid (11–20 seconds) and late (21–30 seconds) post visual stimulus exposure. The magnitude of the NVC response (Δmean) was measured for each distinct region and presented as an absolute change (Δcm/s) and relative change from baseline (Δ%) (Figure 5).

## Statistical analysis protocol

Statistical analysis was completed using statistical software SPSS (IBM statistics software version 26). All normally distributed data are presented as mean ± standard deviation (SD). When the assumption of normal distribution was violated, data are presented as median (IQR; 25^th^-75^th^ percentile). Two core tests were utilised within our statistical analyses: (1) Repeated measures analysis of variance (ANOVA) and (2) Two-way repeated measures ANOVA. The precise protocol and considerations taken during application of each test are detailed below. A report of the full statistical report is provided in supplementary material.

All baseline cardiorespiratory/cerebrovascular measures, arterial blood gases and NVC metrics were first investigated for normal distribution using a combination of Shapiro-Wilk assessment and visual inspection of Q-Q plots. When data satisfied the assumption of normal distribution a repeated-measures ANOVA with Bonferroni post-hoc assessment was performed. The variable of interest was used as the dependent variable and experimental condition used as a within-subjects factor. Mauchly’s test of sphericity was used to determine whether the variance of difference between all combinations of the within-subjects factor was equal. When sphericity was violated a Greenhouse-Geisser or Huynh-Feldt adjustment was performed relative to the estimated Epilson (ε). When data violated the assumption of normal distribution, a non-parametric Friedman rank test equivalent was performed. When data violated the assumption of normal distribution, a manual Bonferroni adjustment was made to account for multiple comparisons and reduce the risk of deducing a type 1 error. Effect size for repeated-measures ANOVA and Friedman rank test were determined using partial eta squared (N_p_^2^) and Kendall’s W (*W*), respectively. Statistical significance was set at p < 0.05.

A two-way repeated measures ANOVA was used to determine spatial specificity of the hemodynamic response to visual stimulation. Two within-subject factors (experimental condition and vessel) were used whereas the magnitude of the hemodynamic response to visual stimulation was used as the dependent variable. Several assumptions were determined prior to final analysis: 1) no significant outliers in combination of the within-subjects factors, 2) the dependent variable should be normally distributed for each combination of the within-subjects factors and 3) the variance of differences between levels should be equal (examined using Mauchly’s test of sphericity). Results were first interpreted for simple main effects and for an interaction effect. Where p > 0.05 for a vessel x condition interaction effect, main effects were interpreted for the two within-subject factors.

## Results

### Baseline cardiovascular measures

All baseline cardiovascular parameters are presented in Table 1. A full detailed breakdown of the statistical analysis can be found in supplementary material. Significant changes were observed for heart rate (p = 0.023), mean arterial pressure (p = 0.033), systolic blood pressure (p = 0.003) and diastolic blood pressure (p = 0.005). *Post hoc* multiple comparisons for between gas and location differences are provided in Table 1.

### Baseline respiratory measures

All baseline respiratory parameters are presented in Table 1. A full detailed breakdown of the statistical analysis can be found in supplementary material. Significant changes were observed for V̇_TI_ (p < 0.001), V̇_I_ (p = 0.002), S_p_O_2_ (p < 0.001), P_ET_O_2_ (p < 0.001) and P_ET_CO_2_ (p < 0.001). R_R_ was unchanged across conditions (p = 0.330). *Post hoc* multiple comparisons for between gas and location differences are provided in Table 1.

### Baseline cerebrovascular measures

All baseline cerebrovascular parameters are presented in Table 1. A full detailed breakdown of the statistical analysis can be found in supplementary material. No significant differences were observed for MCA_v_ (p = 0.488), MCA_cvc_ (p = 0.472), MCA_sys_ (p = 0.446), MCA_dia_ (p = 0.315), PCA_v_ (p = 0.257), PCA_cvc_ (p = 0.392) & PCA_sys_ (p = 0.404,). However, significant changes were observed for MCA_PI_ (p = 0.014), PCA_PI_ (p = 0.005), PCA_dia_ (p = 0.018) and S_c_O_2_ (p < 0.001)). *Post hoc* multiple comparisons for between gas and location differences are provided in Table 1.

### Arterial blood gases and pH

Arterial blood gas and acid-base data are illustrated in Figure 2. A full detailed breakdown of the statistical analysis can be found in supplementary material. Significant changes were observed for P_a_O_2_ (p < 0.001), P_a_CO_2_ (p < 0.001), Hematocrit (p < 0.001) and Hemoglobin (p < 0.05). ${\text{HCO}}_{3}^{-}$ and base excess showed the same pattern of response as PaCO_2_ (p < 0.05 respectively). No significant changes were observed for Arterial pH (p = 0.261). *Post hoc* multiple comparisons for between gas and location differences are provided Figure 2.

### Region-specific hemodynamic response

An illustration of the localised hemodynamic response is shown in Figure 3. A full detailed breakdown of the statistical analysis can be found in supplementary material. Main effects were found for vessel insonation at 1130 m (p < 0.001). The magnitude of the hemodynamic response during visual stimulus was significantly greater within the PCA, compared with the MCA, during normoxia (p < 0.001) and iso-Hx (p < 0.001) conditions. In addition, main effects were found for vessel insonation at WM2 (p < 0.001). The magnitude of the hemodynamic response during visual stimulus was significantly greater within the PCA, compared with the MCA, during normoxia (p < 0.001) and iso-hyperoxia (p < 0.001) conditions. Finally, main effects were found found for vessel insonation at WM9 (p < 0.001). The magnitude of the hemodynamic response during visual stimulation was significantly greater within the PCA, compared with the MCA, during normoxia (p < 0.001) and iso-hyperoxia (p < 0.001) conditions.

### Neurovascular coupling across conditions

An illustration of the NVC response is shown in Figures 4 and 5. A full detailed breakdown of the statistical analysis can be found in supplementary material. No significant differences (p > 0.05) in NVC response magnitude were observed for ΔMean, ΔPeak, ΔtAUC, ΔAcute NVC response, ΔMid NVC response or ΔLate NVC response.

## Discussion

### Key findings

The principal findings are that neither (1) removal of the hypoxic vasodilatory stimulus at high altitude, nor (2) acute and sustained exposure to 3800 m affect the NVC response of the PCA in healthy participants. An illustration of the stability of the NVC responses across all conditions for each participant is shown in Figure 6.

### Changes in cardiorespiratory and cerebrovascular measures

Acute and sustained exposure to high altitude induced a systemic and central hypoxia evidenced by reductions in PaO_2_ (Figure 2), SpO_2_, P_ET_O_2_ and S_c_O_2_ (see Table 1). To mitigate the risk of hypoxic insult, a series of integrative cardiorespiratory and cerebrovascular adaptations are initiated and were observed in our participants. The reduction in PaO_2_ is detected by the carotid body and gives rise to the peripheral respiratory chemoreflex. O_2_-sensitive type I glomus cells housed within the carotid body increase afferent signalling to the respiratory control centres.^38–43^ The net result is an increase in ventilatory drive, which partially restores O_2_ levels. This response is known as the hypoxic ventilatory response and is evidenced herein by increases in inspiratory tidal volume (V_TI_) and minute ventilation (V̇_I_) following acute exposure and sustained residency at high altitude (Table 1). A similar respiratory phenotype is observed following acute exposure to Iso-Hx at 1130 m (Table 1). No further increases in V̇_I_ were observed between days 2 and 9 at 3800 m. This is likely a result of the temporal heterogeneity in ventilatory acclimatisation to chronic hypoxia and acid-base homeostasis within the sample population.

Acute exposure to high altitude elicited an immediate tachycardia,^44–47^ reflecting sympathetic activation. When compared with normoxia at 1130 m, there was an immediate increase in heart rate following acute Iso-Hx exposure at 1130 m and on day 2 at high altitude with no significant differences found on day 9 at high altitude (see Table 1). The mechanisms that drive this cardiac response can largely be attributed to a shift in sympatho-vagal balance following hypoxic activation of the peripheral chemoreceptors.^44–47^

Blood pressure responses to high altitude exposure vary across research expeditions and depend upon 1) the net interaction between hypoxic vasodilation and sympathetic vasoconstriction of systemic vessels, 2) absolute altitude of exposure, 3) individual physiological responses to high altitude and 4) duration of stay at altitude.^46,47^ When compared with normoxia at 1130 m, our results show an increase in diastolic and mean arterial pressure following acute exposure to high altitude with no significant differences observed by day 9 at high altitude (Table 1). Moreover, no changes in systolic blood pressure were observed during acute exposure to and residency at high altitude (Table 1). The increase in blood pressure parameters following acute exposure to high altitude agrees with previous literature which examined correlative changes in blood pressure and acute mountain sickness following acute and prolonged exposure to 3700 m in healthy men.^48^

No statistically significant differences were found for indices of cerebral blood velocity (MCAv and PCAv) across all experimental conditions despite numerical increases in mean values (Table 1). This was unexpected as acute increases in cerebral blood velocity following exposure to high altitude and hypoxia are well-recognised.^1,5,9,49^ Stability in resting cerebral blood velocity might suggest that the interaction between hypoxic vasodilation and hyperventilation-induced hypocapnic vasoconstriction are in equilibrium such that the net effect on cerebral blood flow is unchanged. Notwithstanding, the relative increase in MCAv and PCAv when comparing normoxia (1130 m) and Poi-Hx (WM2) is 9.93% and 19.01%, respectively. A sustained increase of 7.66% and 13.79% for MCAv and PCAv, respectively, was observed when comparing normoxia (1130 m) and Poi-Hx (WM9). The magnitude of the MCAv response mimics previous reports at this altitude.^31^ This observation also highlights potential regional differences in sensitivity within the cerebral blood flow response to high altitude exposure.

### Changes in arterial blood gases and pH with acute and chronic high altitude exposure

The observed changes in arterial blood gases highlight the integrative physiological adaptation to high altitude and mirror previous work from our group.^29^ Acute exposure to high altitude elicited an immediate reduction in PaO_2_ (Figure 2(a)). The respiratory chemoreflex response to hypoxia (i.e., hypoxic ventilatory response) is evidenced by reductions in PaCO_2_ on days 2 and 9 at high altitude (Figure 2(b)). The stepwise reductions in PaCO_2_ between days 2 and 9 despite a fixed high altitude stimulus illustrate plasticity within the respiratory control chemoreceptor-neural axis. Following chronic exposure to hypoxia (as observed during high altitude exposure), there is an increase in both O_2_-sensitivity at the level of the carotid body as well as enhanced central integration within the respiratory control centres.^43,50–55^ The net effect is an increase in ventilatory output for a given PaO_2_ stimulus. We infer evidence of ventilatory acclimatisation to sustained hypoxia with concurrent reductions in PaCO_2_ at 3800 m (Table 1).

Renally-mediated metabolic compensation is elicited owing to the profound respiratory response detailed above. A relative metabolic acidosis is facilitated through the renal system and involves significant reductions in arterial [ ${\text{HCO}}_{3}^{-}$ ] through enhanced excretion in urine (Figure 2(c)).^33,56,57^ This integrative respiratory-renal interaction maintains arterial pH within narrow limits (Figure 2(e)). Lastly, chronic exposure (WM9) to high altitude elicited an increase in both hematocrit and hemoglobin concentration (Figures 2(f) and (g)). This response arises in part due to dehydration and a time-dependent increase in erythropoiesis. This protective mechanism acts to increase circulatory O_2_ carrying capacity, mitigating the risk of hypoxemia.

### Region-specific cerebral blood velocity responses to intermittent visual stimulus

As expected, the cerebral blood velocity response to intermittent visual stimuli was vessel-specific. The magnitude of the hemodynamic response was greater within the PCA compared with the MCA across all experimental conditions (Figure 3). The physiological explanation for the stimulus-dependent hemodynamic response relates to the perfusion territory of both vessels. At a broad level, the MCA perfuses regions of the frontal and temporal lobes, whereas the PCA perfuses posterior regions of the brain, including the occipital lobe, where visual processing occurs.^58^

### NVC response following acute removal of systemic and central hypoxia at high altitude

An important consideration when examining the role of high altitude exposure on NVC is the degree to which hypoxic vasodilation might preserve NVC by countering the influence of multiple cerebral vasoconstrictors (hypocapnia, alkalosis). Hypoxic vasodilation is mediated by a series of erythrocyte-signalling pathways facilitated by the allosteric shift from their relaxed (‘R’) to active (‘T’) state.^3,12^ Deoxyhemoglobin serves to increase nitric oxide (NO) bioavailability through S-nitrosohemoglobin-dependent bioactivity, deoxyhemoglobin-mediated reduction of nitrite to NO and ATP-mediated activation of endothelial nitric oxide synthase.^3,12,59^ Laboratory-based investigations demonstrated mild NVC impairment following acute hyperventilation-induced hypocapnia and alkalosis.^60^ Unlike the high altitude literature, laboratory investigations by Bader et al (2021) and Szabo et al (2011) were not confounded by the competing effects of hypoxia.

The extent to which hypoxia protects NVC function at high altitude is unknown. Hypoxia serves to increase NO bioavailability through a series of pathways described above. NO is an integral modulator and signalling molecule involved within the NVC response. Using hyperoxic inspiratory gas mixtures, our investigation acutely removed the systemic and central hypoxic stimulus on days 2 and 9 at high altitude. We reasoned that this experimental condition would alleviate the hypoxic vasodilation (and subsequent increase in NO bioavailability), and hence facilitate a pro-vasoconstrictive state at high altitude through relative hypocapnia, and allow us to examine the extent to which hypoxia-dependent vasodilation protects NVC function at high altitude. As mentioned previously, lab-based investigations showed that progressive hypocapnia blunts the NVC response in humans.^60^ Importantly, indices of peripheral (P_ET_O_2_, SpO_2_) and cerebral oxygenation (S_c_O_2_) were comparable between normoxia at 1130 m and iso-Hyperoxia challenges on days 2 and 9 at high altitude (Table 1). This observation provides assurance that the competing vasodilatory stimulus at high altitude was acutely removed. However, as shown in Figures 4 and 5, no differences were found when comparing NVC indices across conditions. This observation suggests that hypoxic vasodilation does not protect against NVC impairment in the background of a high altitude -induced relative hypocapnia.

### NVC response during acute and sustained high altitude exposure

The stability in NVC following exposure to high altitude is congruent with previous comparable literature which employed transcranial Doppler ultrasound for the assessment of NVC at high altitude.^29–31^ The previous publications employed incremental^29,30^ and acute^31^ ascent profiles reaching peak altitudes of 4240 m^29,30^ and 3800 m.^31^ NVC was assessed using a variety of stimulus paradigms ranging from visual stimulation^29,31^ to region-specific cognitive tasks^30,31^ while insonating the PCA^29,31^ and the ACA/MCA,^30^ respectively. The rate of ascent within each incremental study^29,30^ was slow, allowing sufficient time for full acclimatisation and metabolic compensatory mechanisms to occur throughout ascent, evidenced by a stable arterial pH with progressive ascent to 4240 m.^29^ Arterial blood draws were not performed by Lefferts et al., (2020), but based upon similarities with ascent profile to Zouboules et al. (2018), one could reason that participants were fully acclimatized to high altitude. Caldwell et al., (2017) incorporated a rapid ascent profile, akin to the ascent profile used herein. However, NVC measurements were recorded on days 3 and 7 following high altitude residency. Arterial blood gases were not recorded during this expedition to characterise arterial oxygenation and pH status at the time of NVC assessment. It is likely that the time delay (3 days) between initial exposure and NVC assessment was sufficient to allow full metabolic compensation of respiratory-induced alkalosis, given our previous reports.

Our hypothesis was based on our understanding of how acid-base disturbances affect cerebrovascular function. Alterations in pH can affect cerebrovascular tone in a direction-dependent manner.^23,24,61,62^ For example, respiratory-induced alkalosis is a profound cerebral vasoconstrictor and routinely observed with rapid ascent to high altitude due to hypoxic ventilatory response-mediated hypocapnia. Under normoxic conditions, respiratory-induced alkalosis induces an immediate reduction in cerebral blood flow.^22,35,60,63^ There is still some debate as to whether this vascular reactivity is driven through pH- and/or CO_2_-mediated mechanisms.^27^ Changes in cerebrovascular tone and consequently cerebral blood flow are achieved through functional manipulation of membrane bound ion channels/receptors, signalling molecules and intracellular enzymes within vascular smooth muscle and endothelial cells.^27,28,61,64–66^ At the point of data collection, only one laboratory-based study had demonstrated that hyperventilation-induced respiratory alkalosis impaired NVC in human participants.^60^ Our hypothesis explored whether similar observations to those made by Bader et al. (2021) and Szabo and colleagues (2011) in a laboratory setting would be found consequent to the respiratory-induced hypocapnia and alkalosis following acute exposure to high altitude.

Our investigation attempted to overcome the temporal issues raised within each of the previous high altitude publications by employing an acute ascent profile (Δ3200 m in approximately 5–6 h) with NVC assessment completed ≤24 h of high altitude exposure. We hypothesized that metabolic compensation of the respiratory-induced alkalosis would not have occurred within this timeframe. This methodological approach would allow us to characterise the pre- vs. post-acclimatisation effects on NVC indices. As shown in Figures 4 and 5, no NVC impairment was observed between experimental conditions. A possible explanation for this observation is evident within the arterial blood analysis (Figure 2). Group-averaged data reveals that arterial pH was statistically compensated at the point of NVC measurement on days two and nine of high altitude exposure. The rapid correction of pH by means of increased bicarbonate excretion within 24 h was unexpected,^36^ but there is considerable heterogeneity in these respiratory-renal responses within 24 h of ascent.

Therefore, our study did not assess NVC against the backdrop of respiratory alkalosis as initially intended. However, a follow-up study by our research group utilised an acute stepwise hyperventilation strategy to achieve progressive hypocapnia and subsequent acute respiratory alkalosis.^35^ NVC in response to intermittent visual stimulation was measured through the PCA at each hypocapnic stage. Given the acute nature of the hyperventilation, it is likely that NVC was assessed against the background of an acute respiratory-induced alkalosis. The results demonstrated small but significant effects of stepwise hypocapnia on the peak NVC response, with no impairment within the mean and/or tAUC NVC response. Similarly, Szabo et al., (2011) found hypocapnic-induced impairment of the peak NVC response to visual stimulation.

Overall, these investigations offer mixed reports on the effects of systemic pH disturbance on NVC performance. Of interest, both laboratory studies demonstrated deleterious effects of hyperventilation-induced hypocapnia on the peak NVC response. However, the impairment observed in Bader et al., (2021) was minor and although observed at -5 Torr (P_ET_CO_2_), it was not further impaired at -10 Torr. Interestingly, CO_2_ within the hypercapnic range has been shown to induce NVC impairment.^67^ Given our growing mechanistic understanding for the temporal involvement of feedforward and feedback pathways within the NVC response,^19,68^ it is possible that stressors, including pH disturbances, might influence specific components of the NVC response rather than affect the magnitude of the response in its entirety.

### Summarising the effects of high altitude exposure on NVC

Overall, the current study and the existing literature suggests that NVC remains intact throughout early and late high altitude exposure. In reconciling the existing state of the literature, two schools of thought can be proposed. The first suggests that there is no interaction between high altitude as a stressor and the physiological pathways integral to NVC function in healthy participants. The second view highlights the utmost importance of NVC to neurophysiological function despite the profound environmental and physiological stress which accompanies high altitude exposure. Other facets of cerebral blood flow regulation (e.g., cerebrovascular reactivity and/or autoregulation) showcase significant vulnerability to high altitude exposure, which demonstrates that cerebral blood flow control mechanisms are not impervious to manipulation.^8,69,70^ However, NVC shows remarkable stability in the face of high altitude and inspired gas-mediated stress. The stability in NVC might be accredited to the milieu of signalling pathways involved within the NVC response, as well as myriad acclimatization processes in haematological, ventilatory and renal systems.

### Additional study limitations

The principal findings are applicable to NVC assessment within the PCA during acute and sustained blood gas challenges. The application of transcranial Doppler ultrasound facilitates assessment of NVC responses within the large conduit cerebral arteries. Whether these observations extend to global brain regions and/or the deeper cerebral microvascular network is unknown. However, to address this would require more advanced neuroimaging techniques which are neither readily available nor portable for the purposes of high altitude expedition research. In addition, transcranial Doppler ultrasound does not provide measurement of underlying vessel calibre. Therefore, there is an inherent risk of misinterpreting the vasodilatory response across blood gas challenges, which may induce vasomotion of conduit cerebral vessels. However, the largest component of vasodilation during NVC stimuli are likely downstream arterioles, proximal to the neuronal pool of interest, as evidenced by the increase in velocity measured in the PCA conduit during application of vasodilatory gas stimuli at baseline and during visual stimulation eliciting NVC. Thus, unlike studies assessing cerebrovascular reactivity, where transcranial Doppler ultrasound utilization has important caveats, studies on NVC are likely better, assessing the effects of downstream arteriolar dilation on an index of flow in the insonated conduit vessel (i.e., PCA).

## Conclusions

In conclusion, the NVC response to intermittent photic stimulation, measured via transient increases in the PCAv, remains unchanged despite (a) removal of the hypoxic vasodilatory stimulus at high altitude and (b) both rapid exposure to and residency at 3800 m. This work highlights remarkable functional stability within the neurovascular network, despite exposure to profound acute and sustained blood gas stressors.

## Supplemental Material

sj-pdf-1-jcb-10.1177_0271678X251322348 - Supplemental material for Characterising the protective vasodilatory effects of hypobaric hypoxia on the neurovascular coupling response Supplemental material, sj-pdf-1-jcb-10.1177_0271678X251322348 for Characterising the protective vasodilatory effects of hypobaric hypoxia on the neurovascular coupling response by Jack K Leacy, David P Burns, Nicholas G Jendzjowsky, Connor Braun, Brittney A Herrington, Richard JA Wilson, Tyler D Vermeulen, Glen E Foster, Alexander J Rosenberg, Garen K Anderson, Caroline A Rickards, Eric F Lucking, Ken D O’Halloran and Trevor A Day in Journal of Cerebral Blood Flow & Metabolism
