# Supplementary material for: Characterizing the protective vasodilatory effects of hypobaric hypoxia on the neurovascular coupling response
Source: J Cereb Blood Flow Metab. 2025 Mar 13;45(7):1293–309. doi: 10.1177/0271678X251322348 (PMC11907632; doi:10.1177/0271678X251322348)
Supplement: sj-pdf-1-jcb-10.1177_0271678X251322348 - Supplemental material for Characterising the protective vasodilatory effects of hypobaric hypoxia on the neurovascular coupling response [file sj-pdf-1-jcb-10.1177_0271678X251322348.pdf]

Submitted to: *Journal of Cerebral Blood Flow and Metabolism*

Subject: Original article

Title: **Characterising the protective vasodilatory effects of hypobaric hypoxia on the neurovascular coupling response**

Authors:
[1]Jack K. Leacy
[1]David P. Burns,
[2,3]Nicholas G. Jendzjowsky,
[2]Connor Braun,
[2]Brittney A. Herrington,
[2,4]Richard J. A. Wilson,
[5]Tyler D. Vermeulen,
[5]Glen E. Foster,
[6,7]Alexander J. Rosenberg,
[6]Garen K. Anderson,
[6]Caroline A. Rickards,
[1]Eric F. Lucking,
[1]Ken D. O'Halloran,
[1,4,8]Trevor A. Day

Affiliations:
[1]Department of Physiology, School of Medicine, College of Medicine and Health, University College Cork, Cork, Ireland
[2]Hotchkiss Brain institute, University of Calgary, Calgary, Alberta, Canada
[3]Respiratory and Critical Care Medicine and Physiology, The Lundquist Institute for Biomedical Innovation at Harbor-UCLA Medical Centre, Torrance, CA, USA
[4]Physiology and Pharmacology, Cumming School of Medicine, University of Calgary, Calgary, Alberta Canada
[5]School of Health and Exercise Sciences, Faculty of Health and Social Development, University of British Columbia Okanagan, British Columbia, USA
[6]Cerebral and Cardiovascular Physiology Laboratory, School of Biomedical Sciences, University of North Texas Health Science Centre, Texas, USA
[7]Physiology Department, Midwestern University, Dower Grove, IL, USA
[8]Department of Biology, Faculty of Science and Technology, Mount Royal University, Calgary, Alberta, Canada

Correspondence: Professor Trevor A. Day
Department of Biology, Faculty of Science and Technology,
Mount Royal University,
Calgary, Alberta, Canada
Telephone: (403)440-5961
Email: tday@mtroyal.ca

Running title: High Altitude and Neurovascular Coupling

Subject area: Cerebrovascular physiology, neurobiology, environmental physiology

FULL STATISTICAL REPORT

Baseline cardiovascular measures

All baseline cardiovascular parameters are presented in Table 1. Heart rate was different across conditions (X^2 (5) = 13.095, p=0.023, W = 0.218). Heart rate increased when comparing normoxia (1130m) with iso-Hx (1130m) and poi-Hx (3800m Day 2, WM2) (p=0.034 and p=0.034, respectively). Mean arterial pressure was different across conditions (X^2 (5) = 17.810, p=0.033, W = 0.297). Mean arterial pressure increased when comparing normoxia (1130m) with poi-Hx and iso-Hyperoxia (WM2) (p=0.032 and p=0.005, respectively). Systolic blood pressure was different across conditions (X^2 (5) = 17.095, p=0.003, W = 0.298). Systolic blood pressure increased when comparing normoxia (1130m) with iso-Hx (1130m) (p=0.007). Diastolic blood pressure was different across conditions ($F_{(2.093, 23.021)}$ = 6.626, p=0.005, n_p^2 = 0.376). Diastolic blood pressure increased when comparing normoxia (1130m) with iso-Hx (1130m), poi-Hx & iso-Hyperoxia (WM2 collectively) (p=0.016, p=0.024 & p=0.009, respectively).

Baseline respiratory measures

All baseline respiratory parameters are presented in Table 1. R_R was unchanged across conditions (X^2 (5) = 5.762, p=0.330, W = 0.096). $\dot{V}_{TI}$ was different across conditions ($F_{(2.272, 24.994)}$ = 8.191, p<0.001, n_p^2 = 0.427). $\dot{V}_{TI}$ increased when comparing normoxia (1130m) with iso-Hx (1130m, p=0.038), poi-Hx (WM2, p=0.037), iso-Hyperoxia (WM2, p=0.026), poi-Hx (WM9, WM9, p<0.001) and iso-Hyperoxia (WM9, p=0.008). Moreover, statistical differences (p=0.037) were observed between poi-Hx (WM9) and iso-Hx (1130m). $\dot{V}_I$ was different across conditions (X^2 (5) = 19.429, p=0.002, W = 0.324). $\dot{V}_I$ increased when comparing normoxia (1130m) with poi-Hx (p=0.011) and iso-Hyperoxia (p=0.001) on WM2 and poi-Hx (p=0.048) and iso-Hyperoxia (p=0.048) on WM9. S_pO_2 was different across conditions (X^2 (5) = 51.190, p<0.001, W = 0.853).

S_pO_2 was lower when comparing normoxia (1130m) with iso-Hx (1130m, $p<0.001$) & poi-Hx (WM2, p=0.003). Iso-Hyperoxia (WM2) was higher when compared with iso-Hx (1130m, $p<0.001$), poi-Hx (WM2, $p<0.001$) and poi-Hx (WM9, p=0.002). Iso-Hyperoxia (WM9) was higher when compared with iso-Hx (1130m, $p<0.001$) and poi-Hx (WM2, p=0.011). $P_{ET}O_2$ was different across conditions (X^2 (5) = 51.190, $p<0.001$, W = 0.853). As expected, $P_{ET}O_2$ was higher when comparing normoxia with iso-Hx (1130m, $p<0.001$), poi-Hx (WM2, $p<0.001$) & poi-Hx (WM9, p=0.016). Similarly, Iso-Hyperoxia $P_{ET}O_2$ (WM2) was higher when compared with iso-Hx (1130m, $p<0.001$) & poi-Hx (WM2, p=0.002). Iso-Hyperoxia $P_{ET}O_2$ (WM9) was higher when compared with iso-Hx (1130m, p=0.016) & poi-Hx (WM2, p=0.002). $P_{ET}CO_2$ was different across conditions (X^2 (5) = 49.095, $p<0.001$, W = 0.818). Poi-Hx $P_{ET}CO_2$ (WM2) was lower compared with iso-Hx (1130m, p=0.016). Poi-Hx $P_{ET}CO_2$ (WM9) was lower compared with normoxia (1130m, $p<0.001$), iso-Hx (1130m, $p<0.001$) & iso-Hyperoxia (WM2, p=0.002). Iso-Hyperoxia $P_{ET}CO_2$ (WM9) was lower compared with normoxia (1130m, p=0.002) & iso-Hx (1130m, $p<0.001$).

Baseline cerebrovascular measures

All baseline cerebrovascular parameters are presented in Table 1. No main effect was found for MCA_v ($F_{(1.884, 18.839)}$ = 0.728, p=0.488, n_p^2 = 0.068), MCA_{cvc} ($F_{(1.923, 19.226)}$ = 0.771, p=0.472, n_p^2 = 0.072), MCA_{sys} ($F_{(1.790, 17.896)}$ = 0.814, p=0.446, n_p^2 = 0.075), MCA_{dia} ($F_{(2.031, 20.307)}$ = 1.227, p=0.315, n_p^2 = 0.109), PCA_v ($F_{(1.865, 20.518)}$ = 1.448, p=0.257, n_p^2 = 0.116), PCA_{cvc} ($F_{(1.735, 19.082)}$ = 0.952, p=0.392, n_p^2 = 0.080) & PCA_{sys} ($F_{(2.250, 24.752)}$ = 0.964, p=0.404, n_p^2 = 0.081). MCA_{PI} was different across conditions ($F_{(2.205, 22.052)}$ = 5.024, p=0.014, n_p^2 = 0.334). The change in MCA_{PI} was lower when comparing normoxia (1130m) with poi-Hx (WM2, p=0.013) and iso-Hyperoxia (WM2, p=0.010). PCA_{PI} was different across conditions (X^2(5) = 16.571, p=0.005, W = 0.276).

The change in PCA_{PI} was lower when comparing normoxia (1130m) with poi-Hx (WM2, p=0.016) and iso-Hyperoxia (WM2, p=0.023). PCA_{dia} was different across conditions ($F_{(2.381,\ 26.192)}$ = 4.353, p=0.018, n_p^2 = 0.284). PCA_{dia} was higher when comparing normoxia (1130m) with poi-Hx (WM2, p=0.038). S_cO_2 was different across conditions (X^2(5) = 38.698, p<0.001, *W* = 0.704). S_cO_2 was higher when comparing iso-Hyperoxia (WM2) with iso-Hx (1130m, p<0.001), poi-Hx (WM2, p=0.001) & poi-Hx (WM9, p=0.001). Iso-Hyperoxia S_cO_2 (WM9) was higher when compared with iso-Hx (1130m, p=0.002), poi-Hx (WM2, p=0.006) & Poi-Hx (WM9, p=0.008).

Arterial blood gases and pH

Arterial blood gas and acid-base data are illustrated in Figure 2. P_aO_2 was different across time-points (X^2(2) = 20.667, p<0.001, *W* = 0.861). As expected, P_aO_2 was higher when comparing 1130m (1130m) with WM2 (p<0.001) and WM9 (p=0.013). P_aCO_2 was different across time-points ($F_{(1.132,\ 12.456)}$ = 18.556, p<0.001, n_p^2 = 0.628). P_aCO_2 was lower when comparing WM2 (p=0.002) and WM9 (p=0.003) with 1130m. Moreover, P_aCO_2 was further reduced when comparing WM2 and WM9 (p=0.015). HCO_3^- and base excess showed the same pattern of response as $PaCO_2$. Arterial pH was stable across time-points ($F_{(2,20)}$ = 1.439, p=0.261, n_p^2 = 0.126). Hematocrit was different across time-points ($F_{(2,\ 22)}$ = 27.073, p<0.001, n_p^2 = 0.711). Hematocrit was higher when comparing WM9 with 1130m (p<0.001) and WM2 (p<0.001). Hemoglobin concentration was different across time-points ($F_{(2,\ 20)}$ = 22.202, p<0.001, n_p^2 = 0.689). Hemoglobin concentration levels were higher when comparing WM9 with 1130m (p<0.001) and WM2 (p<0.001).

Region-specific hemodynamic response

No vessel x condition simple main effects were found for 1130m (Δcm/s; $F_{(1,10)}$ = 0.242, p=0.633, n_p^2 = 0.024 & Δ%; $F_{(1,10)}$ = 4.131, p=0.070, n_p^2 = 0.292). Main effects

found an effect for vessel insonation (Δcm/s; $F_{(1,10)} = 65.082$, $p<0.001$, $n_p^2 = 0.867$ & Δ%; $F_{(1,10)} = 187.767$, $p<0.001$, $n_p^2 = 0.949$). The magnitude of the hemodynamic response during visual stimulus was significantly greater within the PCA, compared with the MCA, during normoxia ($p<0.001$) and iso-Hx ($p<0.001$) conditions at 1130m. No vessel x condition simple main effects were found for 3800m day 2 (Δcm/s; $F_{(1,11)} = 0.775$, $p=0.397$, $n_p^2 = 0.066$ & Δ%; $F_{(1,11)} = 1.985$, $p=0.187$, $n_p^2 = 0.153$). Main effects found an effect for vessel insonation (Δcm/s; $F_{(1,11)} = 71.116$, $p<0.001$, $n_p^2 = 0.866$ & Δ%; $F_{(1,11)} = 71.667$, $p<0.001$, $n_p^2 = 0.867$). The magnitude of the hemodynamic response during visual stimulus was significantly greater within the PCA, compared with the MCA, during normoxia ($p<0.001$) and iso-hyperoxia ($p<0.001$) conditions at WM2. No vessel x condition simple main effects were found for WM9 (Δcm/s; $F_{(1,10)} = 0.099$, $p=0.759$, $n_p^2 = 0.010$ & Δ%; $F_{(1,10)} = 0.367$, $p=0.558$, $n_p^2 = 0.035$). Main effects found an effect for vessel insonation (Δcm/s; $F_{(1,10)} = 64.923$, $p<0.001$, $n_p^2 = 0.867$ & Δ%; $F_{(1,10)} = 94.954$, $p<0.001$, $n_p^2 = 0.905$). The magnitude of the hemodynamic response during visual stimulation was significantly greater within the PCA, compared with the MCA, during normoxia ($p<0.001$) and iso-hyperoxia ($p<0.001$) conditions at WM9.

Neurovascular coupling across conditions

NVC response magnitude for PCAv was not different across conditions for ΔMean (Δcm.s; $F_{(2.383, 26.218)} = 1.457$, $p=0.251$, $n_p^2 = 0.117$ & Δ%; $F_{(2.14, 23.54)} = 0.917$, $p=0.419$, $n_p^2 = 0.077$), ΔPeak (Δcm.s; $F_{(2.243, 24.671)} = 1.424$, $p=0.260$, $n_p^2 = 0.115$ & Δ%; $F_{(2.01, 22.07)} = 1.21$, $p=0.317$, $n_p^2 = 0.099$), ΔtAUC (Δcm.s^2; $F_{(2.297, 22.969)} = 1.641$, $p=0.214$, $n_p^2 = 0.141$ & Δ%; $X^2(5) = 9.857$, $p=0.079$, $W = 0.179$), ΔAcute NVC response (Δcm.s; $X^2(5) = 6.532$, $p=0.258$, $W = 0.119$ & Δ%; $X^2(5) = 5.048$, $p=0.410$, $W = 0.084$), ΔMid NVC response (Δcm.s; $X^2(5) = 5.130$, $p=0.400$, $W = 0.093$ & Δ%; $X^2(5) = 10.273$, $p=0.068$, W

= 0.187), ΔLate NVC response (Δcm.s; $F_{(5, 50)}$ = 1.749, p=0.141, n_p^2 = 0.149 & Δ%; $F_{(2.207, 22.069)}$ = 2.267, p=0.123, n_p^2 = 0.185).
